# Comparative transcriptomic and proteomic analysis reveals common molecular factors responsive to heat and drought stresses in sweetpotaoto (*Ipomoea batatas*)

**DOI:** 10.3389/fpls.2022.1081948

**Published:** 2023-01-19

**Authors:** Wei Tang, Mohamed Hamed Arisha, Zhenyi Zhang, Hui Yan, Meng Kou, Weihan Song, Chen Li, Runfei Gao, Meng Ma, Xin Wang, Yungang Zhang, Zongyun Li, Qiang Li

**Affiliations:** ^1^ Xuzhou Institute of Agricultural Sciences in Jiangsu Xuhuai District/Sweetpotato Research Institute, Chinese Academy of Agricultural Sciences/Key Laboratory of Biology and Genetic Breeding of Sweetpotato, Ministry of Agriculture and Rural Affairs, Xuzhou, China; ^2^ Institute of Integrative Plant Biology, Jiangsu Key Laboratory of Phylogenomics and Comparative Genomics, School of Life Sciences, Jiangsu Normal University, Xuzhou, China; ^3^ Department of Horticulture, Faculty of Agriculture, Zagazig University, Zagazig, Sharkia, Egypt

**Keywords:** sweetpotato, heat, drought, transcriptome, proteome

## Abstract

**Introduction:**

Crops are affected by various abiotic stresses, among which heat (HT) and drought (DR) stresses are the most common in summer. Many studies have been conducted on HT and DR, but relatively little is known about how drought and heat combination (DH) affects plants at molecular level.

**Methods:**

Here, we investigated the responses of sweetpotato to HT, DR, and DH stresses by RNA-seq and data-independent acquisition (DIA) technologies, using controlled experiments and the quantification of both gene and protein levels in paired samples.

**Results:**

Twelve cDNA libraries were created under HT, DR, and DH conditions and controls. We identified 536, 389, and 907 DEGs in response to HT, DR, and DH stresses, respectively. Of these, 147 genes were common and 447 were specifically associated with DH stress. Proteomic analysis identified 1609, 1168, and 1535 DEPs under HT, DR, and DH treatments, respectively, compared with the control, of which 656 were common and 358 were exclusive to DH stress. Further analysis revealed the DEGs/DEPs were associated with heat shock proteins, carbon metabolism, phenylalanine metabolism, starch and cellulose metabolism, and plant defense, amongst others. Correlation analysis identified 6465, 6607, and 6435 co-expressed genes and proteins under HT, DR, and DH stresses respectively. In addition, a combined analysis of the transcriptomic and proteomic data identified 59, 35, and 86 significantly co-expressed DEGs and DEPs under HT, DR, and DH stresses, respectively. Especially, top 5 up-regulated co-expressed DEGs and DEPs (At5g58770, C24B11.05, Os04g0679100, BACOVA_02659 and HSP70-5) and down-regulated co-expressed DEGs and DEPs (AN3, PMT2, TUBB5, FL and CYP98A3) were identified under DH stress.

**Discussion:**

This is the first study of differential genes and proteins in sweetpotato under DH stress, and it is hoped that the findings will assist in clarifying the molecular mechanisms involved in sweetpotato resistance to heat and drought stress.

## Introduction

Heat and drought stresses tend to occur together under natural conditions, and are considered the most prevalent stresses in terrestrial environments (Prasad et al., 2011; Vile et al., 2012). These abiotic stresses adversely affect plant growth, resulting in delayed growth, abnormal flowering, photosynthetic disorders, and oxidative stress, amongst other effects (Jagtap et al., 1998; Mittler, 2006; Silva et al., 2010; Carmo-Silva et al., 2012). The physiological adaptations of plants to the DH stress differ from those in response to the single stressors of HT and DR (Shulaev et al., 2008). Rizhsky et al. (2002) found that tobacco responds specifically to the DH stress. For example, genes that are up-regulated under HT or DR stress may be down-regulated under the combined stressors. Recent studies have further confirmed that plants possess unique molecular and physiological response mechanisms to the DH stress (Safronov et al., 2017; Woldesemayat et al., 2018). It is well-documented that HT and DR stresses can damage both the physiology and metabolism of plant cells. These stressors can directly or indirectly damage the cell membrane, destroying its structure and causing the leakage of critical molecules and ions, as well as damaging the functioning of the cell (Tommasino et al., 2018). Reactive oxygen species (ROS) are produced in the electron transport chain between Photosystems I and II (PSI and PSII, respectively) in the chloroplast. When plants are subjected to HT and DR stresses, excess excitation energy is produced, leading to ROS accumulation and potential damage to the photosystem (Demmig-Adams and Adams, 1992). Rubisco is a carboxylase in the C3 photosynthetic pathway. Decreases in photosynthesis associated with HT and DR stresses are closely related to the inactivation of Rubisco. As Rubisco reduces plant carbon assimilation under HT and DR stresses, there is an excess of excitation energy for the light response and a consequent increase in ROS production (Law and Crafts-Brandner, 1999).

Advances in biotechnology have allowed the identification of molecular factors involved in stress tolerance, elucidating the plant underlying mechanisms to stress. To date, numerous stress-associated molecules and pathways have been identified. One of the key pathways is the abscisic acid (ABA) pathway which regulates the plant response to DR. This pathway can produce ROS and increase the levels of cytosolic calcium, together with activating ion channels to trigger stomatal closure (Flexas and Medrano, 2002; Safronov et al., 2017). The response to HT relies on various pathways, all of which involve the participation of heat shock proteins (HSPs) (Wang et al., 2004). Under HT stress, most HSPs are able to avoid misfolding and consequent aggregation, allowing them to refold during recovery. The mechanisms responsible for this vary between the HSP families (Rampino et al., 2009). For instance, HSP60 and HSP70 bind to protein intermediates, preventing aggregation, and HSP101 can undergo renaturation and restoration of function after earlier denaturation (Katiyar-Agarwal et al., 2003).

Sweetpotato (*Ipomoea batatas* (L.) Lam.) is an annual or perennial plant belonging to the *Convolvulaceae* family, which is the top 4 crop in China along with rice, wheat and corn (Yang et al., 2017). It is a tropical food crop and has the advantages of high yield and the ability to withstand stress, compared with rice, wheat, and other major crops (Loebenstein and Thottappilly, 2009). To date, many genes linked to abiotic stress tolerance have been identified and cloned in sweetpotato, such as *IbDREB* (Kim et al., 2010), *IbOr* (Kim et al., 2013), *IbP5CR* (Liu et al., 2014), *IbGI* (Tang et al., 2017), *IbTC* (Kim et al., 2019), *IbMYB116* (Zhou et al., 2019), and *IbSUT* (Wang et al., 2020). Furthermore, RNA-seq has been applied to sweetpotato transcriptome study under single-stress conditions, including salt (Arisha et al., 2020a), drought (Yang et al., 2018; Arisha et al., 2020b), and cold (Ji et al., 2017; Ji et al., 2019; Xie et al., 2019; Ji et al., 2020) stress using second- and third-generation sequencing on Illumina platforms. Therefore, understanding the strategies adopted by stress-tolerant sweetpotato can assist in the elucidation of the molecular mechanisms underlying stress tolerance in crops.

To explore the mechanisms underlying tolerance to HT, DR, and their combination DH in *Ipomoea batatas*, we used both transcriptomic and proteomic analyses in controlled experiments, measuring the expression of genes and proteins in paired samples. Using Illumina HiSeq and data-independent acquisition (DIA) technology, we identified differentially expressed genes (DEGs) and proteins (DEPs) under HT, DR, and DH stresses. The roles of these genes in response to the stressors were analyzed and are discussed below.

## Plant materials and methods

The purple-flash sweetpotato Xuzishu 8, a popular cultivar with a high anthocyanin content, high yield, and good taste and drought tolerance (Yan et al., 2022), was used for transcriptomic and proteomic sequencing. The cultivar was bred by the Xuzhou Institute of Agricultural Sciences in Jiangsu Xuhuai District, Xuzhou City, China. The seedlings of Xuzishu 8 were planted in the loamy and fluvo-aquic field (pH 8.2) in Xuzhou sweetpotato research center (34°16’N, 117°17’E) on June 10^th^. After 60 d, the plants were dug out and grown under hydroponic culture using Hoagland solution. Then the plants were treated by HT (42°C), DR (1 liter of 30% PEG6000 Hoagland solution), and DH (42°C and 1 liter of 30% PEG6000 Hoagland solution) in the growth chamber with 90 μM/m^2^/s of light intensity and 50% relative humidity under long day (LD) condition (16 h light/8 h dark). The fully opened third unifoliate samples and storage roots samples with three biological replications were collected during the treatments from 0.5, 1, 3, 6, 12, 24, and 48 h for transcriptomic and proteomic analysis. Control specimens grown under hydroponic culture using Hoagland solution were put in another growth chamber with 25°C, 90 μM/m^2^/s of light intensity and 50% relative humidity under LD condition, and biological samples in triplicate were also collected at the same time points. Samples were immediately frozen in liquid nitrogen and then maintained at -80°C.

### Total RNA extraction and experimental design

Total RNA free of genomic DNA was extracted using a Total RNA Extraction Kit (Generay, China), following the provided protocols. Purity and quality were assessed by 1% agarose gel electrophoresis and spectrophotometry (Nanophotometer, IMPLEN, CA, USA) using the A260/A280 and A260/A230 ratios. RNA concentrations were determined with a Qubit^®^ RNA Assay Kit in Qubit^®^2.0 Fluorometer (Life Technologies, CA, USA) and integrity was measured with the RNA Nano 6000 Assay Kit of the Agilent Bioanalyzer 2100 system (Agilent Technologies, CA, USA). We then mixed equal amounts of RNA samples from leaves and tuberous roots at each time point under heat, drought, and their co-stresses to construct four sequencing libraries, namely, HT, DR, DH, and CK (control). Each library was constructed and analyzed in triplicate resulting in 12 sequencing libraries for 12 pooled samples.

### cDNA library construction and transcriptome sequencing

cDNA libraries were constructed using 5 μg of total RNA per pool with the VAHTSTM mRNA-seq V2 Library Prep Kit for Illumina^®^, following the provided protocol, and index codes were used for sequence identification in individual samples. Poly-T oligo-attached magnetic beads were used to purify mRNA which was fragmented with divalent cations at high temperature in 5x VAHTSTM First Strand Synthesis Reaction Buffer. The first-strand cDNA was synthesized with a random hexamer primer and M-MuLV Reverse Transcriptase (RNase H-) and second-strand cDNA was synthesized using DNA polymerase I and RNase H. Exonucleases and polymerases were used to convert overhangs to blunt ends. The 3′-ends of the fragmented DNA were adenylated and ligation of the adaptor was performed before library preparation. The fragments were purified using the AMPure XP system (Beckman Coulter, Beverly, USA) to allow preferential selection of 150-200 bp fragments which were then treated with 3 μl of USER Enzyme (NEB, USA) for 15 min at 37°C and then for 5 min at 95°C. PCR used Phusion High-Fidelity DNA polymerase, Universal PCR primers, and Index (X) primer and the products were purified using AMPure XP, with quality assessment using an Agilent Bioanalyzer 2100 system. The libraries were quantified and pooled, and paired-end sequencing was conducted on HiSeq XTen sequencers (Illumina, San Diego, CA, USA).

### Analysis of transcriptomic data

After the conversion of the sequencer-produced images into raw nucleotide reads, the quality of the reads was assessed using FastQC (http://www.bioinformatics.babraham.ac.uk/projects/fastqc/) and filtering was conducted using Trimmomatic (Bolger et al., 2014). The clean reads were then aligned to the sweetpotato reference genome (Yang et al., 2017) with HISAT2 (Kim et al., 2015). The expression of genes was defined by the abundance of their transcripts, which were quantified using StringTie (Pertea et al., 2015), calculating the Fragments Per Kilobase per Million (FPKM) values of the protein-coding genes and lncRNAs in the samples. DEGs were identified using DESeq2 (Love et al., 2014) using the criteria of FDR <0.05 and difference multiple |log_2_ FC| >1.

### Quantitative real-time PCR

The samples for transcriptome sequencing were also used for qRT-PCR, which was conducted on a StepOne Plus real-time PCR system (Applied Biosystems) using SYBR Green Master Mix (Roche, Germany). Gene expression was calculated using the comparative C(T) method (Schmittgen and Livak, 2008). The reference gene was *tublin* of *Ipomoea batatas*. Three technical and two biological replicates were used for each analysis. Primer sequences are listed in **Table S1**.

### Protein preparation

Proteins were extracted from the same 12 pooled samples used for transcriptome sequencing. The plant material was pulverized in liquid nitrogen and further homogenized in pyrolysis solution (7M urea, 2% SDS, 0.1% PMSF, 65 mM DTT) with ultrasonication. Supernatants were centrifuged (14 000 rpm, 30 min, 4 °C) and the protein contents were measured by the BCA method (Walker, 1994). After that, the proteins were reduced and alkylated using 50 ul DTT (1 h, 55 °C) and 5 ul of 20 mM iodoacetamide (IAA; 1 h, room temperature, in the dark), respectively. The proteins were then precipitated for 2 h with 300 ul of precooled acetone and hydrolyzed with trypsin (Promega) overnight. The resulting peptides were dissolved in buffer A (buffer A: 20 mM ammonium formate aqueous solution, adjusted to pH 10.0 by ammonia water) and separated using a reverse column (XBridge C18 column, 4.6 mm x 250 mm, 5 um) connected to an Ultimate 3000 system (Waters Corporation, MA, USA) using 5% to 45% solution B (20 mM ammonium formate with 80% ACN, adjusted with ammonia to pH 10.0) for separation. Fractions were lyophilized before analysis.

### Establishment of DDA database and DIA data collection

The peptides were redissolved in an aqueous solution of 0.1% formic acid aqueous solution and applied to an LC-MS/MS Orbitrap Lumos mass spectrometer with an online nanojet ion source (Thermo Fisher Scientific, Ma, USA). This spectrometer operates in data-dependent acquisition (DDA) mode and switches automatically between MS and MS/MS acquisition. The raw DDA data were analyzed using a Spectronaut X (Biognosys AG, Switzerland) with default settings, producing an initial target list. The *Ipomoea batatas* database was searched by the Spectronaut together with a database of contaminants derived by trypsin digestion. The material was suspended in 30 ul of aqueous 0.1% formic acid solution, of which 9 ul was mixed with 1 ul 10 x iRT peptide segment. Sample separation was performed by nano-LC and analyzed by on-line electrospray ionization tandem mass spectrometry for data-independent acquisition (DIA).

### Proteome data analysis

The raw DIA data were analyzed using the Spectronaut X using default parameters and dynamic iRT retention time Data were extracted by Spectronaut X using extensive mass calibration. The optimal extraction window dependent on the iRT calibration and stability of the gradient was determined by Spectronaut Pulsar (Kim et al., 2018). A cutoff Q-value (FDR) of 1% for the levels of precursors and proteins was set. A setting of “mutated” was used for decoy generation; this resembles “scrambled” while only applying random numbers of residue positions (min=2, max=length/2). Precursors that passed through the filters were used for quantification, with the top three filtered peptides passing the 1% Q-value threshold being used to determine the numbers in the major groups. After Student’s *t*-test, the Benjamini and Hochberg method was applied, and DEPs were filtered using the criteria of FDR <0.05 and difference multiple |log_2_FC| ≥0.585 (Liu et al., 2020).

### Functional annotation

Protein and gene annotations were determined using the NCBI non-redundant (NR), Gene Ontology (GO), and Kyoto Encyclopedia of Genes and Genomes (KEGG) databases. The numbers of DEGs or DEPs were used to calculate p-values (<0.05) and q-values (<0.05) using the total number of genes or proteins as backgrounds. The p-value is a measure of the significance of enrichment, while the q-value controls for the FDR. P-values were determined by Fisher’s exact test and the q-values were measured using the “qvalue” package in R.

### Protein and RNA correlation analyses

Co-expression of genes and proteins was analyzed by the determination of correlation coefficients, conducted in R (version 3.5.1). Maps with nine quadrants were created to illustrate alterations in gene and protein expression in the transcriptomic and proteomic data, respectively, with the map showing the quantification and enrichment of the genes or proteins in each region of the map. Genes and proteins and DEGs and DEPs in the transcriptome and proteome, respectively, were assessed separately, and Venn diagrams were used to illustrate the data.

## Results

### Transcriptome sequencing, quality filtering, and assembly

Separate sequencing of 12 cDNA libraries was conducted on the Illumina HiSeq 2000 platform, resulting in a total of 85,378,804,200 raw reads. When the adaptor sequences, low-quality, and short reads, and rRNA reads were removed, 82,750,736,262 clean reads remained (**Table S2**). The GC content was 57–66%, Q20 92–97%, and the percentage of unknown nucleotides (N%) was 0%. Then, the clean reads that mapped to the reference genome were categorized into two classes: uniquely mapped reads, namely, reads that mapped to only one position in the reference genome, and multi-position matches, namely, reads that mapped to more than one position in the reference genome (**Table S3**).

### Identification of DEGs

**Figure 1:**
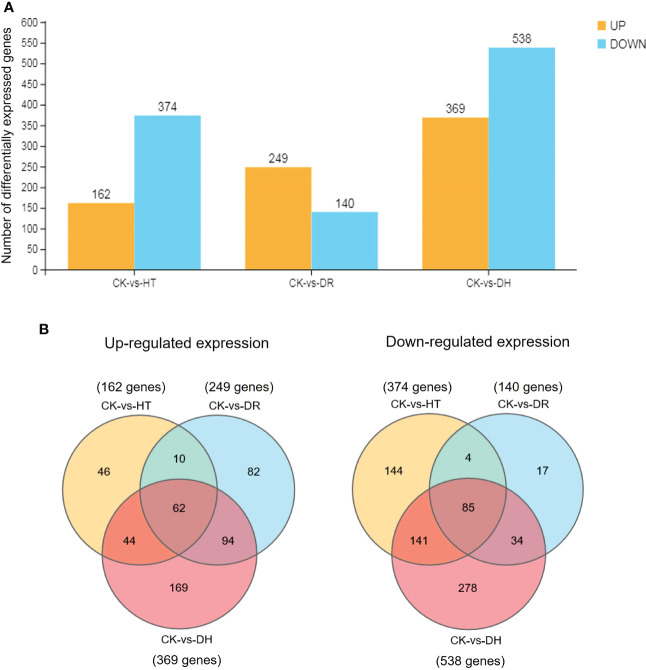
Comparisons of DEGs. **(A)** Number of DEGs under all stress conditions. DEGs were defined by FDR <0.05 and |log_2_ of fold change (FC)| >1. Yellow indicates up-regulation and blue indicates down-regulation. **(B)** Venn diagram illustrating the up-regulated (left side) and down-regulated (right side) genes under HT, DR, and DH stresses. CK, control; HT, heat stress; DR, drought stress; DH, drought and heat stresses.

The level of gene expression in individual samples was calculated as the FPKM. Using the criteria of FDR <0.05 and |log_2_ of fold change (FC)| >1, 536, 389, and 907 DEGs were identified in response to HT, DR, or DH stress, respectively, in sweetpotato cv. Xuzishu 8, of which 162, 249, and 369 were up-regulated, and 374, 140, and 538 were down-regulated (**Figure 1A**). Venn diagrams were used to highlight overlapping DEGs expressed among HT and DH, DR and DH, as well as HT, DR, and DH stresses. Under HT and DR stresses, there was an overlap of 72 up-regulated and 89 down-regulated genes. Similarly, there were overlaps of 156 up-regulated and 119 down-regulated genes under the combination of DR and DH stresses, and of 106 up-regulated and 226 down-regulated genes under HT and DH stresses. Of the 147 overlapping genes among the DR, HT, and DH stresses, 62 were up-regulated and 85 were down-regulated. Furthermore, top 10 common up- and down-regulated DEGs (**Table 1**) were identified under HT, DR and DH stresses, of which seven up-regulated DEGs including *CHIT1B*, *TPM-1*, *BXL1*, *dnajb6*, *HSP21*, *MLS* and *Os04g0179200*, and one down-regulated DEG *CHX20* were overlapped. In addition, the transcriptome of plants subjected to DH stress contained 169 specifically elevated genes and 278 genes that were specifically reduced by this combined stress (**Figure 1B**).

**Table 1:** Top 10 common up- and down-regulated DEGs among the overlapping genes in HT, DR, and DH.

CK-vs-HT			CK-vs-DR			CK-vs-DH		
Symbol_id	log_2_fc	*q-*value	Symbol_id	log_2_fc	*q-*value	Symbol_id	log_2_fc	*q-*value
Up-regulated								
*TPM-1*	18.445	2.585E-06	*CHIT1B*	20.046	2.357E-19	*CHIT1B*	18.689	9.01E-14
*BXL1*	16.475	9.333E-05	*TPM-1*	19.832	6.101E-18	*TPM-1*	16.908	1.571E-10
*CHIT1B*	15.201	3.913E-05	*BXL1*	12.223	0.0079	*BXL1*	13.147	0.0003
*dnajb6*	11.215	0.0198	*dnajb6*	11.648	0.0024	*dnajb6*	10.954	0.0015
*HSP21*	10.351	4.007E-06	*Os04g0179200*	10.079	7.92E-08	*EDL3*	10.646	4.244E-15
*PR2*	8.785	0.0018	*CHI2*	9.179	3.013E-10	*HSP21*	9.883	9.255E-10
*MLS*	8.092	4.571E-05	*EDL3*	8.951	2.028E-12	*MAN2*	9.513	1.406E-09
*Os04g0179200*	8.044	0.0056	*HSP21*	8.889	3.266E-06	*ICL*	9.055	1.38E-09
*aq_1250*	7.976	0.0037	*aq_1250*	8.631	2.178E-06	*Os04g0179200*	8.946	6.243E-05
*CXE15*	7.860	3.014E-05	*MLS*	8.223	2.942E-17	*MLS*	8.328	9.01E-14
Down-regulated								
*DFRA*	-13.870	3.350E-04	*CHI3*	-12.687	0.0070	*FL*	-17.117	0.0017
*XTH1*	-12.615	8.290E-04	*CHX20*	-12.306	0.0089	*RD22*	-16.871	0.0011
*CHX20*	-12.306	0.0083	*GSPO-A1*	-8.423	4.126E-06	*AED3*	-15.480	1.181E-05
*GSPO-A1*	-10.838	4.118E-06	*AED3*	-7.441	4.341E-05	*BGLU47*	-12.526	0.0035
*At4g28780*	-9.877	5.718E-07	*DFRA*	-7.227	0.0029	*CHX20*	-12.306	0.0081
*CHSE*	-9.646	5.718E-07	*At1g28590*	-7.085	1.821E-04	*CHSE*	-11.072	1.352E-08
*ABP19A*	-9.302	3.010E-08	*AED3*	-7.085	5.768E-04	*At4g28780*	-9.999	2.055E-08
*FL*	-9.230	1.458E-05	*PHO1*	-6.851	3.458E-04	*ABP19A*	-9.993	1.451E-11
*GSTF11*	-9.198	3.516E-04	*LSH1*	-6.849	8.091E-06	*NIR1*	-9.314	1.167E-04
*NIR1*	-8.990	2.554E-05	*ERF003*	-6.731	1.294E-04	*PALA*	-9.264	1.318E-09

### GO and KEGG enrichment analysis

**Figure 2:**
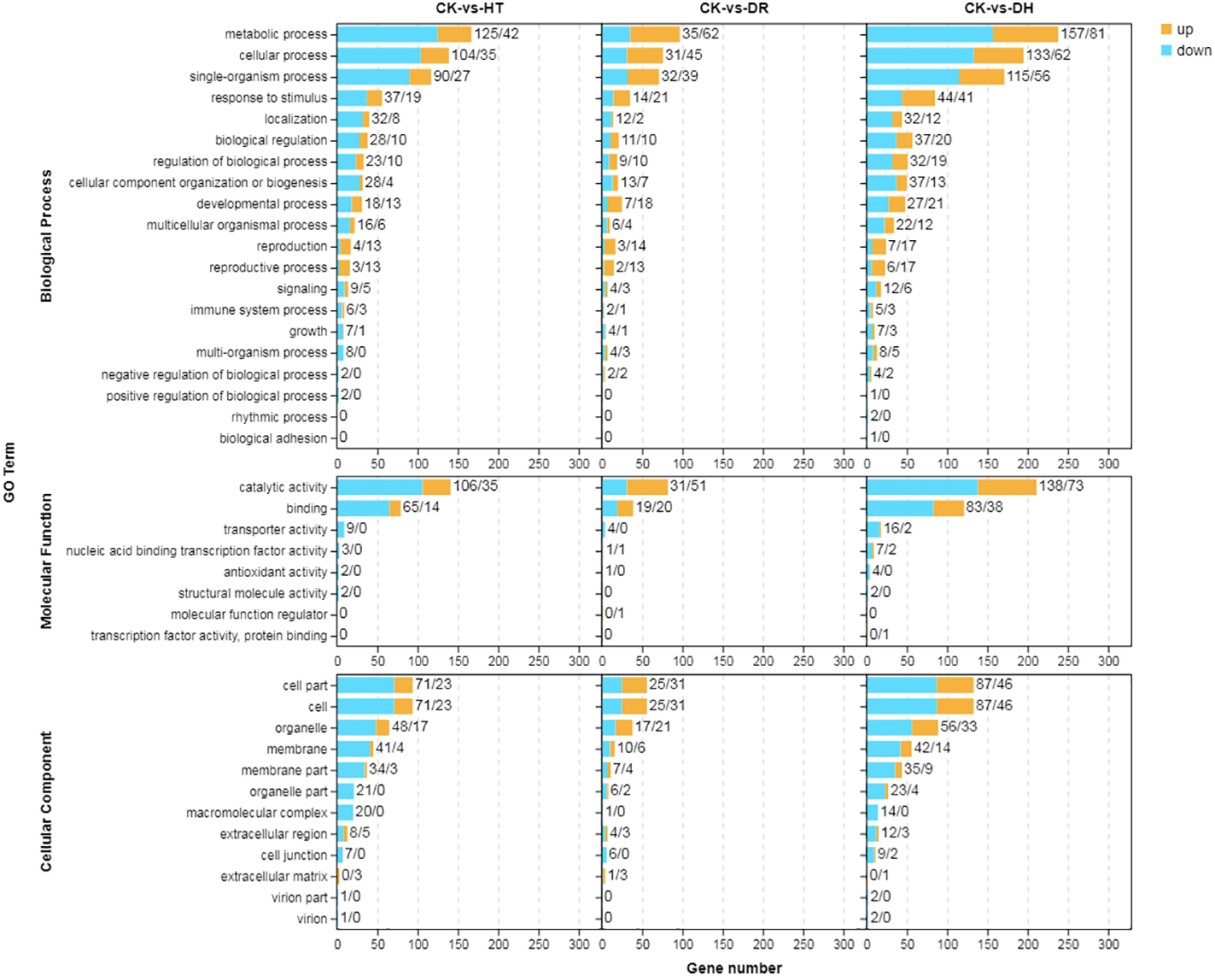
GO annotation of DEGs in response to HT, DR, or DH stress. Yellow indicates up-regulation and blue indicates down-regulation. CK, control; HT, heat stress; DR, drought stress; DH, drought and heat stresses.

GO was used to determine the principal functional classifications of the DEGs. Nearly 54%, 57%, and 55% of DEGs under HT, DR, and DH stresses, respectively, were associated with the ‘biological process’ category. Similarly, 17%, 17%, and 19% of DEGs associated with HT, DR, and DH stresses, respectively, were seen in the ‘molecular function’ classification, and 29%, 26%, and 27%, respectively, were found under ‘cellular component’. Under HT, DR, or DH stress, the top four classes of upregulated and downregulated genes from ‘biological process’ level 2 GO terms were metabolic process (GO:0008152), cellular process (GO:0009987), single-organism (GO:0044699), and response to stimulus (GO:0050896). In ‘molecular function’, the top classes were catalytic activity (GO:0003824) and binding (GO:0005488) under all conditions, while in ‘cellular component’, the top three classes of up-regulated and down-regulated genes were cell (GO:0005623), cell part (GO:0044464) and organelle (GO:0043226). The DR stress condition showed greater numbers of up-regulated than down-regulated genes in most of the GO categories, while there were fewer up-regulated genes under HT or DH stress (**Figure 2**).

**Figure 3:**
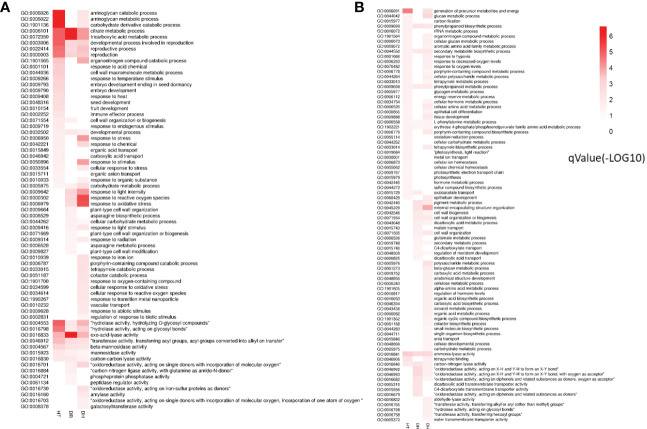
Heatmap of the *q*- values for GO categories showing DEG enrichment under HT, DR and DH stresses compared with CK. **(A)** Functional enrichment analysis of up-regulated DEGs under HT, DR, and DH stresses. **(B)** GO terms of down-regulated DEGs under HT, DR, and DH stresses. The color scale ranges from white (no DEGs), light-red (DEGs with low *q*-values) to red (DEGs with high *q*-values). HT, heat stress; DR, drought stress; DH, drought and heat stresses.

The GO annotations that showed significantly higher levels of DEG enrichment (*q* < 0.05) under the HT, DR, and DH treatments (**Figure 3A**) included aminoglycan catabolic and metabolic process (GO:0006026 and GO:0006022), organonitrogen compound catabolic process (GO:1901565), carbohydrate metabolic process (GO:0044262 and GO:0005975), citrate and tricarboxylic acid metabolic process (GO:0006101 and GO:0072350), beta-mannosidase activity (GO:0004567), hydrolase activity (GO:0004553 and GO:0016798), and oxo-acid-lyase activity (GO:0016833), amongst others. Categories showing significant enrichment in down-regulated genes included phenylpropanoid biosynthetic and metabolic process (GO:0009699 and GO:0009698), secondary metabolite biosynthetic process (GO:0044550), cell wall biogenesis (GO:0042546), ammonia-lyase activity (GO:0016841), and carbon-nitrogen lyase activity (GO:0016840) (**Figure 3B**). Overall, these findings suggest that sweetpotato shares a “cross-tolerance” in response to heat, drought, and their combination. However, an opposite trend was found between DR and HT stresses, with the gene expression trend under DH stress being closer to that of HT stress.

Of the significantly enriched GO terms (**Table S4**), the DEGs specifically up-regulated in DH were mainly found in the “environmental response process”, “RNA process”, and “compound catabolic process” categories. “Environmental response process” included the annotations cellular response to oxidative stress and reactive oxygen species (GO:0034599 and GO:0034614), response to iron ion (GO:0010039), response to transition metal nanoparticle (GO:1990267), response to oxygen-containing compound (GO:1901700), response to ethylene (GO:0009723), and oxidoreductase activity (GO:0016730 and GO:0016703). “RNA process” contained regulation of transcription (GO:0006355), regulation of nucleic acid-templated transcription (GO:1903506), and regulation of RNA biosynthetic and metabolic process (GO:2001141 and GO:0051252). “Compound catabolic process” included porphyrin-containing compound catabolic process (GO:0006787), aromatic compound catabolic process (GO:0019439), cellular nitrogen compound catabolic process (GO:0044270), amylase activity (GO:0016160), and galactosyltransferase activity (GO:0008378). GO enrichment analysis of the potential functions of the genes down-regulated under DH stress identified over 30 categories with significant enrichments (*q* < 0.05) under DH stress, such as cell wall organization (GO:0071555), anatomical structure development (GO:0048856), beta-glucan metabolic process (GO:0051273), cellulose metabolic process (GO:0030243), flavonoid metabolic process (GO:0009812), and water transmembrane transporter activity (GO:0005372).

**Figure 4:**
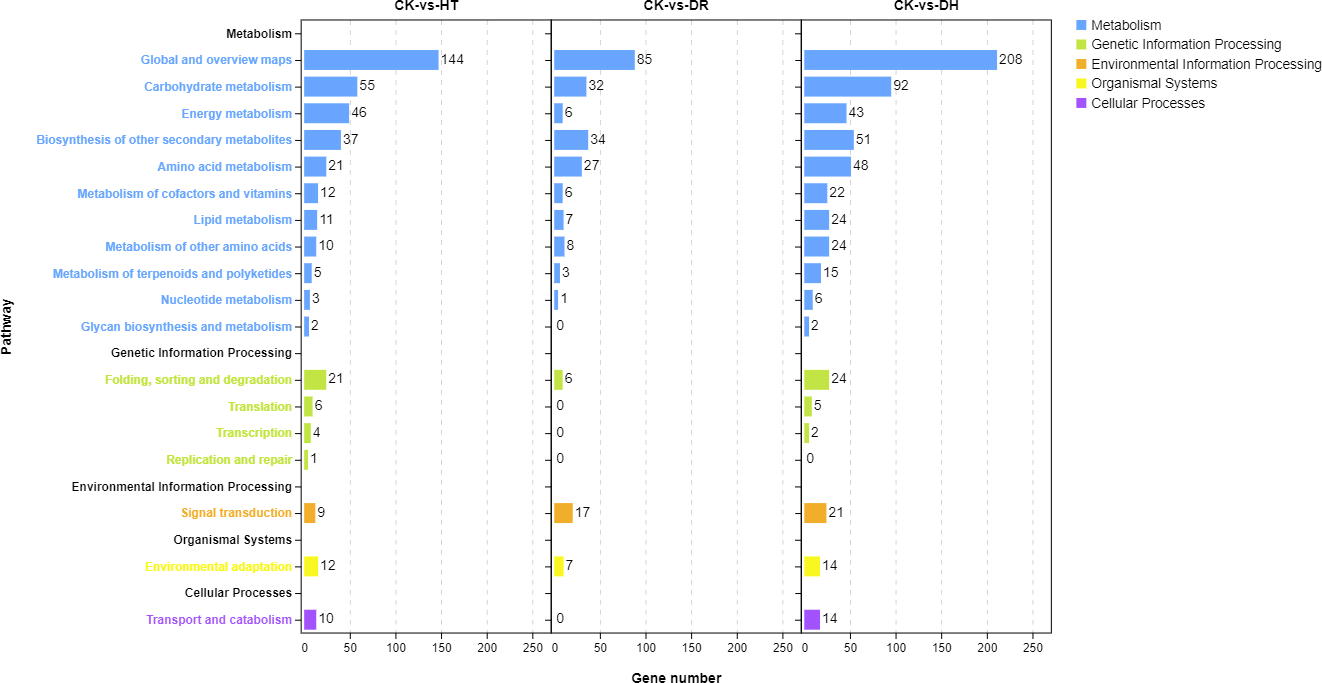
KEGG analysis of DEGs in the HT, DR, and DH stress groups compared with CK. CK, control; HT, heat stress; DR, drought stress; DH, drought and heat stresses.

Investigation of pathway enrichment using KEGG (**Figure 4**; **Table S5**) showed that most of the enriched pathways were shared by DEGs under HT, DR, or DH stress. Four hundred and four DEGs from the three groups were associated with 98 different KEGG pathways in 18 clades under five major KEGG categories including metabolism, environmental information processing, genetic information processing, cellular processes, and organismal systems.

**Figure 5:**
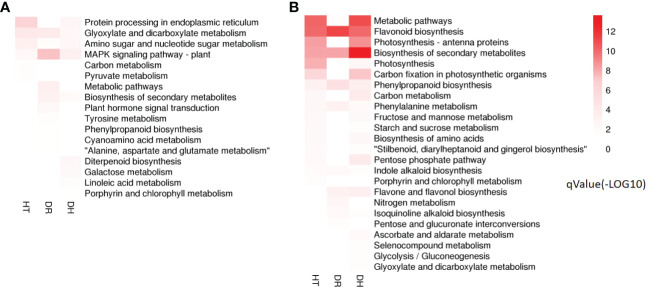
Heatmap of the *q*-value significance of KEGG pathways showing DEG enrichment in response to HT, DR, and DH stresses compared with CK. **(A)** Up-regulated DEGs under HT, DR, and DH stresses. **(B)** Down-regulated DEGs under HT, DR, and DH stresses. The color scale ranges from white (no DEGs), light-red (DEGs with low *q* values) to red (DEGs with high *q* values). HT, heat stress; DR, drought stress; DH, drought and heat stresses.

KEGG analysis was used to identify the pathways showing DEG enrichment (*q* < 0.05). Gene-set enrichment analysis showed that DEGs in the glyoxylate and dicarboxylate metabolism, amino sugar and nucleotide sugar metabolism, and the MAPK signaling pathway were significantly up-regulated in all the stress categories. In addition, specific pathways with DH-associated DEG enrichment were diterpenoid biosynthesis, galactose metabolism, linoleic acid metabolism, and porphyrin and chlorophyll metabolism (**Figure 5A**). Pathways showing down-regulated DEGs tended to be common to all the stress groups and included metabolic pathways, flavonoid biosynthesis, secondary metabolites biosynthesis, phenylpropanoid biosynthesis, phenylalanine metabolism, and indole alkaloid biosynthesis. Four pathways were unique to DH stress, namely, ascorbate and aldarate metabolism, selenocompound metabolism, glycolysis/gluconeogenesis, and glyoxylate and dicarboxylate metabolism (**Figure 5B**). These GO and KEGG analyses suggested that similar pathways were involved in the response to different stress conditions, indicative of cross-tolerance to stress in the sweetpotato. Furthermore, the combination of heat and drought stresses appears to act synergistically to trigger specific biochemical processes and molecular functions, in contrast to the response seen to the individual stressors.

### Proteomics analysis and identification of DEPs

We then analyzed the functions of proteins responding to HT, DR, and DH stresses using proteome sequencing. The DIA method integrates the benefits of the traditional proteomics “shotgun” and selective reaction monitoring/multiple reaction monitoring (SRM/MRM) approaches with absolute quantitative mass spectrometry, and was used to investigate changes in protein expression in response to HT, DR, and DH stresses. Initially, we identified 50 457 precursors, 39 376 peptides, and 9862 proteins from 6189 protein groups in 12 pooled samples (**Figure S1A**). GO analysis showed that these proteins were associated with 48 functional groups including all three GO categories of biological process, cellular component, and molecular function (**Figure S1B**). Under biological process, the highest enrichments were seen in metabolic processes (2451 proteins, 26.30%) followed by cellular processes (1991, 21.36%), and single-organism processes (1597, 17.14%). Under molecular function, the most enriched associations were catalytic activity (2044, 51.25%) and binding activity (1639, 41.10%). The top category under cellular components was cell parts (1678, 25.34%) (**Table S6**).

**Figure 6:**
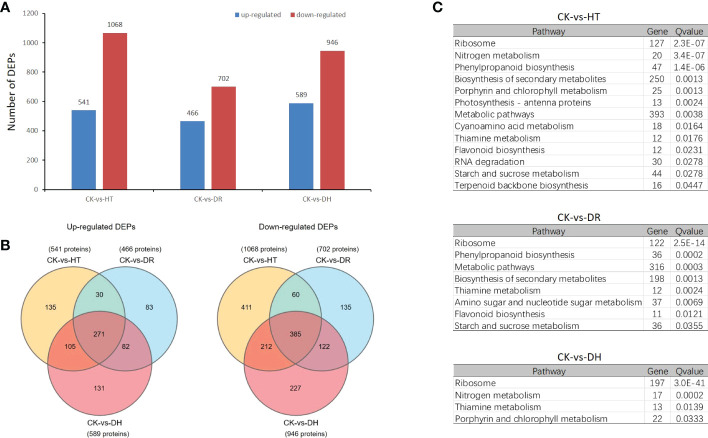
Differentially expressed proteins analysis under HT, DR and DH stresses. **(A)** Numbers of up- and down-regulated DEPs. **(B)** Venn diagrams of DEPs. Left: up-regulated DEPs; right: down-regulated DEPs. **(C)** KEGG pathways showing significant (*q* < 0.05) DEP enrichment for each stress category.

Overall, 1609, 1168, and 1535 DEPs were identified in the HT, DR, and DH groups compared with the CK group, of which 541, 466, and 589 were up-regulated and 1068, 702, and 946 were down-regulated, respectively (**Figure 6A**). A total of 271 up-regulated DEPs were common to the three groups, while 135, 83, and 131 were exclusively up-regulated under HT, DR, and DH stresses. In addition, 385 down-regulated DEPs overlapped between the different groups, while 411, 135, and 227 were specific to the HT, DR, and DH groups, respectively (**Figure 6B**). KEGG analysis of the DEPs under HT stress showed that the four top pathways were “Ribosome” (*q*-value 2.31e-7), “Nitrogen metabolism” (*q*-value 3.36e-7), “Phenylpropanoid biosynthesis” (*q*-value 1.38e-6), and ‘Biosynthesis of secondary metabolites’ (*q*-value 0.00128). The additional nine pathways (*q* < 0.05) included ‘Porphyrin and chlorophyll metabolism’, ‘Photosynthesis - antenna proteins’, ‘Metabolic pathways’, and ‘Cyanoamino acid metabolism’ (**Figure 6C**, upper panel). The pathways identified for DR stress were not only involved in several of the same biological processes as those for HT stress, such as ‘Ribosome’ (*q*-value 2.5e-14), ‘Phenylpropanoid biosynthesis’ (*q*-value 0.0002), ‘Metabolic pathways’ (*q*-value 0.0003), ‘Biosynthesis of secondary metabolites’ (*q*-value 0.0013), ‘Thiamine metabolism’ (*q*-value 0.0024), and ‘Flavonoid biosynthesis’ (*q*-value 0.0121) but also included two specific pathways, namely, ‘Amino sugar and nucleotide sugar metabolism’ (*q*-value 0.0069) and ‘Starch and sucrose metabolism’ (*q*-value 0.0355) (**Figure 6C**, middle panel). The DH-stress group included ‘Ribosome’ (*q*-value 3.0e-41), ‘Nitrogen metabolism’ (*q*-value 0.0002), ‘Thiamine metabolism’ (*q*-value 0.0139), and Porphyrin and chlorophyll metabolism (*q*-value 0.0333) which overlapped with both the HT and DR groups (**Figure 6C**, lower panel).

The top 10 DEPs identified under HT, DR, and DH stresses are shown in **Table 2**. Several up-regulated DEPs are common to each stress, including PER72 which catalyzes the final step in lignin biosynthesis, small heat-shock proteins HSP23.6 involved in processing in the endoplasmic reticulum, ACT domain-containing protein ACR11, actin binding protein VILLIN2 for actin cytoskeleton and stem-specific protein TSJT1. Some stress specific up-regulated DEPs are also found, such as Exo70A1 which is involved in targeted secretion at the plasma membrane under HT stress, guanylate-binding protein GBP2 under DR stress, cold and drought-regulated protein CORA under DH stress, amongst others. As in HT stress, HSP23.6 and two proteins (PER72 and PNC1) which are key enzymes catalyzing the formation of lignin from monolignol, were also up-regulated under DR stress. In contrast, few down-regulated DEPs were shared between the HT, DR, and DH stress groups. Under HT stress, some proteins associated with metabolite biosynthesis, CYP51G1 (sterol 14-demethylase), TH1 (thiamine biosynthetic bifunctional enzyme), and ANS (anthocyanidin synthase) were down-regulated. Under DR stress, several of the top 10 down-regulated DEPs were found to be involved in ‘biological process’, including the ubiquitin receptor RAD23B, HMGCL which is responsible for the synthesis and degradation of ketone bodies, starch branching enzyme I SBE1, and ANT17 which is involved in the process of anthocyanin synthesis. Some specific top 10 down-regulated DEPs are found under DH stress, such as the novel plant SNARE protein NPSN11, plasmodesmata callose-binding protein PDCB2, and ABC transporter G family member ABCG22, amongst others. Although the same ‘Flavonoid biosynthesis’ pathway is annotated in each stress category, three different down-regulated DEPs (ANS, ANT17, and AN3) were specifically involved in the anthocyanin synthesis.

**Table 2:** Top 10 up- and down-regulated proteins in HT, DR, and DH identified by DIA.

CK-vs-HT			CK-vs-DR			CK-vs-DH			
Symbol_id	log_2_fc	*q-*value	Symbol_id	log_2_fc	*q-*value		Symbol_id	log_2_fc	*q-*value
Up-regulated									
VLN2	15.582	0.0014	VLN2	15.752	0.0039		CORA-like	16.859	0.0429
At5g45670	15.426	0.0304	TSJT1-like	14.965	0.0010		VLN2	15.753	0.0023
TSJT1-like	14.805	0.0011	BPI	10.200	0.0052		TSJT1-like	15.647	0.0040
HSP23.6	6.418	0.0113	ACR11	6.460	0.0234		AVP1	12.735	0.0353
ACR11	5.902	0.0282	At5g58770	6.446	0.0194		HSP23.6	6.113	0.0123
PER72	5.188	0.0070	HSP23.6	6.206	0.0189		ACR11	6.064	0.0389
EXO70A1	4.969	0.0032	GBP2	6.133	0.0163		At5g58770	5.710	0.0178
MBD11	4.920	0.0198	SRC1-like	5.952	0.0325		PER72	5.163	0.0105
CYP82C3	4.862	0.0211	PER72	5.313	0.0091		MBD11	4.797	0.0222
PARA	4.817	0.0001	PNC1	4.957	0.0225		PARA	4.715	0.0329
Down-regulated									
CYP51G1	-16.437	0.0028	RAD23B	-14.953	0.0032		BRR2B	-15.614	0.0200
BRR2B	-15.696	0.0173	OsI_14861	-14.539	0.0461		RAD23B	-15.063	0.0022
RPT2	-14.852	0.0037	At4g00740	-12.190	0.0026		NPSN11	-15.024	0.0015
SBT1.7	-14.083	0.0436	HMGCL	-4.687	0.0398		ABCG22	-5.410	0.0060
TH1	-12.008	0.0031	URGT2	-4.123	0.0036		AN3	-4.215	3.32e-10
MCM2	-11.492	0.0413	OBERON1-like	-4.113	0.0169		FSH2	-4.159	0.0050
FAD2	-8.193	0.0224	At1g28590	-3.926	0.0158		PDCB2	-3.997	0.0024
ANS	-5.213	0.0312	SBE1	-3.808	2.07e-15		Os03g0108600	-3.984	0.0097
typA	-5.112	3.83e-07	ANT17	-3.801	0.0072		At1g26850	-3.835	0.0007
UGT83A1	-4.633	0.0174	Os03g0108600	-3.725	0.0104		OBERON1-like	-3.669	0.0497

### Correlations of RNA and protein expression

**Figure 7:**
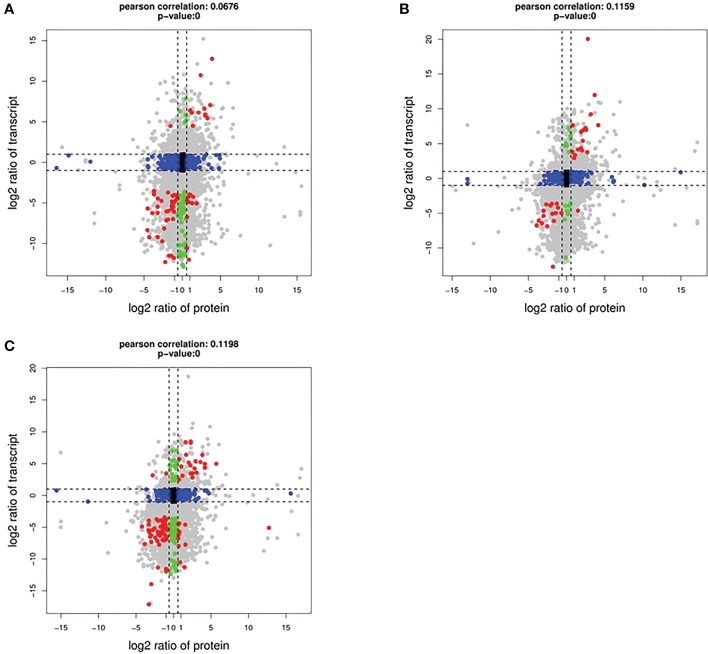
Log_2_ expression ratios in the transcriptome (y-axis) and proteome (x-axis). HT **(A)**, DR **(B)**, and DH **(C)** conditions. Significant alterations in expression are indicated by color: blue, protein; green, transcripts; red, both.

**Figure 8:**
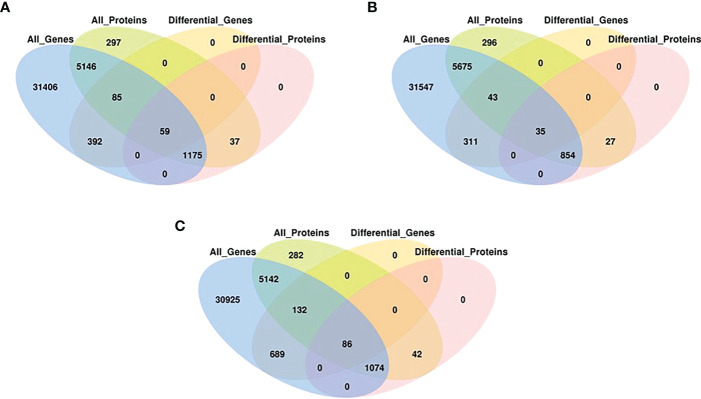
Venn diagrams associations between, all expressed genes, all expressed proteins, DEGs (*q* < 0.05) and DEPs (*q* < 0.05) with stringent criteria under HT **(A)**, DR **(B)**, and DH **(C)** stresses.

To determine the co-expression relationships between genes and proteins under each stress condition, correlations between the transcriptomic and proteomic results were analyzed. Initially, we identified input 38 263 genes and 8891 proteins for HT, 38 465 genes and 9079 proteins for DR, 38 048 genes and 8852 proteins for DH, respectively. The Pearson correlation coefficients of the genes with their encoded proteins were 0.0676 under HT stress, 0.1159 under DR stress, and 0.1198 under DH stress (**Figure 7**). Venn diagrams showed that 6465 co-expressed genes and proteins were present under HT stress, of which 59 were both DEGs and DEPs (**Figure 8A**). In the DR-stress category, 6607 genes were co-expressed with their proteins, and 35 of these were both DEGs and DEPs (**Figure 8B**). In the DH category, there were 6435 co-expressed genes and proteins, of which 86 were both DEGs and DEPs (**Figure 8C**). Thus, we suggest that these co-expressed DEGs and DEPs may play critical roles in the defense against heat, drought, and combined drought and heat stresses in the sweetpotato.

### GO and KEGG analyses of co-expressed DEGs and DEPs

GO analysis of the co-expressed DEGs and DEPs indicated that ‘cellular process’ and ‘metabolic process’ predominated. There were 89, 56, and 232 co-expressed DEGs and DEPs for the HT, DR, and DH stress conditions, respectively, in the “cellular process” category, and 173, 140, and 400 co-expressed DEGs and DEPs, respectively, in ‘metabolic process’. In terms of “cellular component”, co-expressed DEGs and DEPs with ‘cell’ and ‘cell part’ annotations were present under all stress conditions. The highest numbers of co-expressed DEGs and DEPs were seen in the ‘organelle’ category, with 16 and 80 genes and proteins in the DR and DH conditions, respectively. In “molecular function”, the highest number of DEG/DEP co-expression was seen in ‘binding’ “catalytic activity”, with 64, 13, and 132 co-expressed DEGs and DEPs in ‘binding’, and 30, 16, and 44 in ‘catalytic activity’ for HT, DR, and DH stresses, respectively (**Figure S2**).

**Figure 9:**
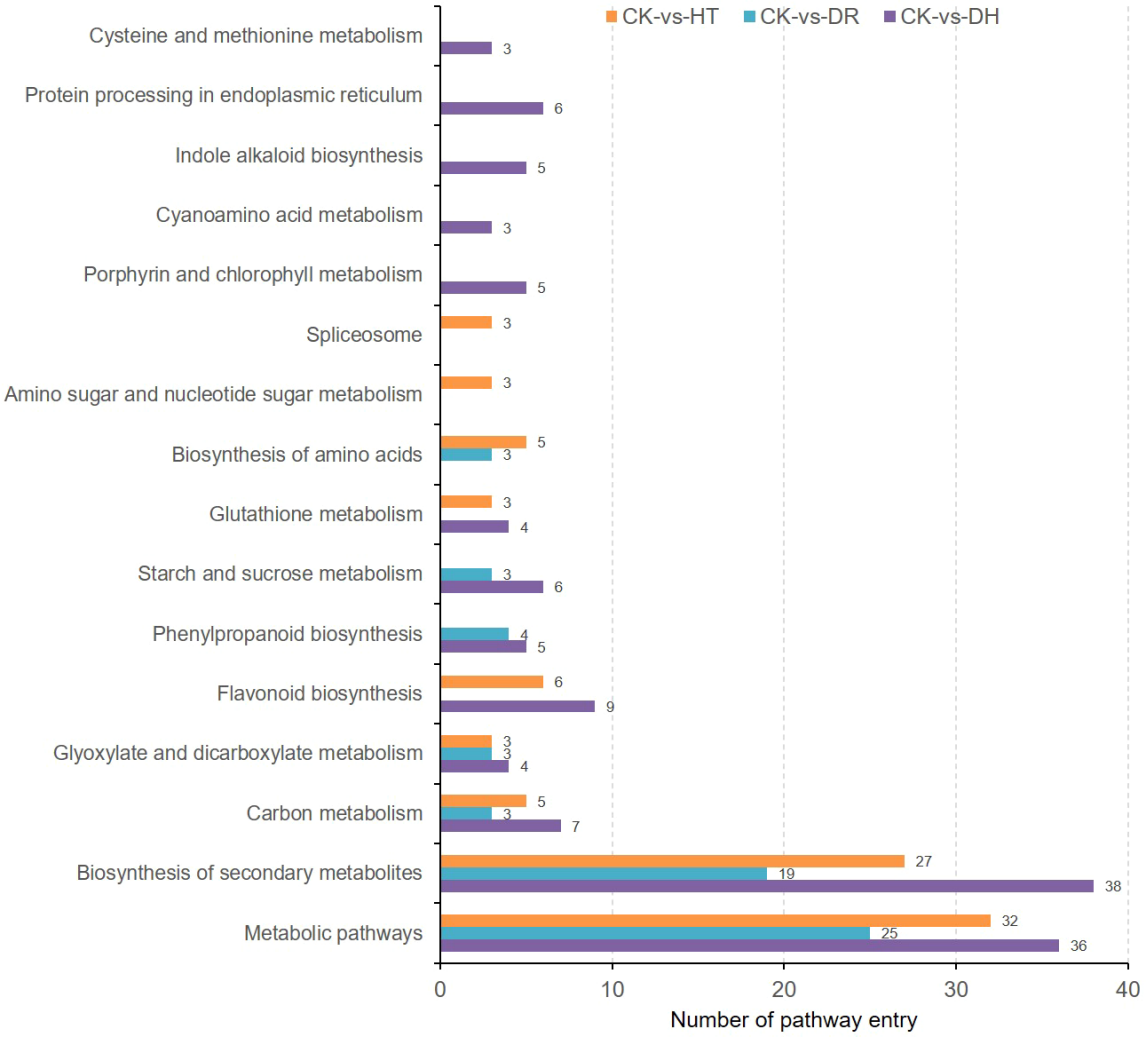
KEGG pathway analysis of co-expressed DEGs and DEPs under HT, DR, and DH stresses.

KEGG analysis showed enrichment of the co-expressed DEGs and DEPs under HT stress in pathways associated with ‘Metabolic pathway’, ‘Biosynthesis of secondary metabolites’, ‘Flavonoid biosynthesis’, ‘Biosynthesis of amino acids’, and ‘Carbon metabolism’. The top enrichment pathways under DR stress were ‘Metabolic pathway’, ‘Biosynthesis of secondary metabolites’, and ‘Phenylpropanoid biosynthesis’, while those under DH stress were ‘Metabolic pathway’, ‘Biosynthesis of secondary metabolites’, ‘Flavonoid biosynthesis’, and ‘Carbon metabolism’. There were also several pathways that were specifically enriched under DH stress, including ‘Protein processing in endoplasmic reticulum’, ‘Porphyrin and chlorophyll metabolism’, and ‘Indole alkaloid biosynthesis’ (**Figure 9**).

### Cross-validation of transcriptomic and proteomic data

**Figure 10:**
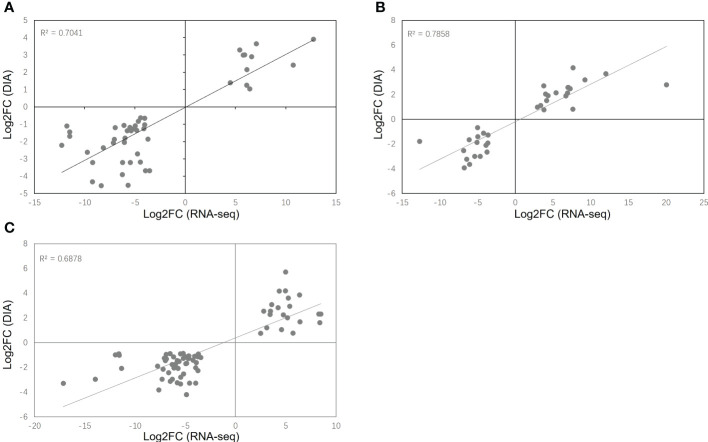
Scatter plots of co-expressed DEGs and DEPs (FDR < 0.05, simultaneously) in the transcriptome and proteome under HT **(A)**, DR **(B)**, and DH **(C)** stresses.

Transcriptomic and proteomic analysis was conducted using RNA-seq and DIA, allowing cross-validation between the two sets of data. A comparison of the significant DEGs and DEPs (FDR < 0.05, simultaneously) showed that 47 DEGs/DEPs were consistently expressed under HT stress, of which 11 were consistently up-regulated and 36 were down-regulated under HT stress. Similarly, there were 34 consistent DEGs/DEPs (18 up-regulated and 16 down-regulated) under DR stress and 72 consistent DEGs/DEPs (21 up-regulated and 51 down-regulated) under DH stress (**Table S7**). The coverage of these DEGs/DEPs against total co-expressed DEGs and DEPs for HT, DR and DH were 2.70%, 2.29% and 4.04%, respectively. Scatter plots were used to illustrate the comparisons between the transcriptome and proteome in the different stress conditions (**Figure 10**) demonstrating DEGs and DEPs that showed consistency between the two data sets. The R-square values for consistent DEGs and DEPs were 0.7041 for HT, 0.7858 for DR, and 0.6978 for DH. These findings indicate strong agreement between the two data sets. Furthermore, to verify the reproducibility and accuracy of the transcriptomic and proteomic data, eight co-expressed DEG and DEP genes were randomly selected for qPCR verification; these were G13246, G14317, G23318, G27744, G33304, G38888, G42670, and G26280. The results showed that the co-expressed DEG and DEP genes from the transcriptomic and DIA data have similar expression profiles under the different stress conditions (**Figure S3**).

## Discussion

**Figure 11:**
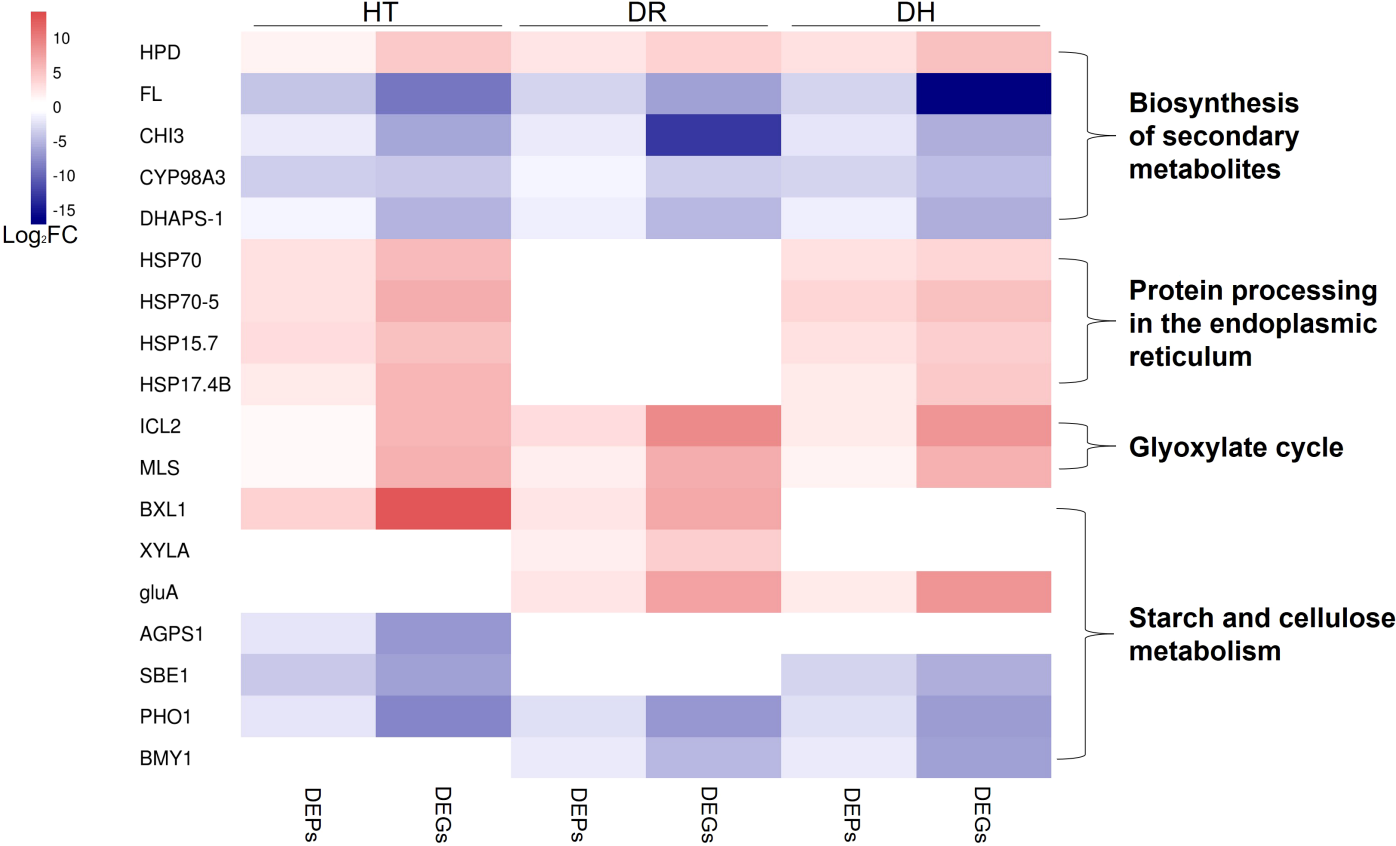
Heatmap of the fold change of DEGs/DEPs (FDR <0.05, simultaneously) in processes of biosynthesis of secondary metabolites, endoplasmic reticulum processing of proteins, glyoxylate cycle, and starch and cellulose metabolism under HT, DR, and DH stresses. The color scale ranges from navy (downregulated DEGs/DEPs), white (no DEGs/DEPs) to firebrick (upregulated DEGs/DEPs). HT, heat stress; DR, drought stress; DH, drought and heat stresses.

Here, genes involved in the responses to HT, DR, and DH stresses were identified by RNA-seq and DIA proteomic analyses. Many of these genes were significantly (**Table S7**) associated with the biosynthesis of secondary metabolites, endoplasmic reticulum processing of proteins, glyoxylate cycle, and starch and cellulose metabolism (**Figure 11**), amongst other processes. This appears to be the first investigation into the molecular effects of abiotic stresses in the sweetpotato using both transcriptomic and proteomic approaches.

### Biosynthesis of secondary metabolites

Plant secondary metabolites (PSM) are key to plant survival under biotic or abiotic stress conditions (Wink, 2016). Most PSMs consist of phenolic acids, flavonoids, terpenoids, steroids, and alkaloids, all of which have been implicated in regulating plant defense mechanisms (Bourgaud et al., 2001). The present study found several types of PSM-associated genes specifically expressed in the HT, DR, and DH groups. One of these was 4-hydroxyphenylpyruvate dioxygenase (HPD) which catalyzed a key step in tocopherol biosynthesis (Dreesen et al., 2018) and was found to be up-regulated in groups of HT, DR, and DH. α-Tocopherol is known to be closely connected to the activity of vitamin E which scavenges ROS and protects the integrity of membranes (Ren et al., 2011). Recently, Kim et al. (2021) found that sweetpotato plants overexpressed with *HPD* enhanced tolerance to various abiotic stresses including DR and elevated in α-tocopherol content compared with non-overexpressed plants. Nevertheless, several genes associated with flavonoid biosynthesis were observed to be down-regulated under all three stress conditions, these included FL (G11743), CHI3 (G29792), CYP98A3 (G25739), and DHAPS-1 (G6925). It has been found that raised temperatures led to reduced flavonoid contents in the bark of the Norway spruce (*Picea abies*) (Virjamo et al., 2014), and the flavonoid content of the bark chlorenchyma was observed to decrease significantly under drought stress, despite rehydration of seedlings. (Zhang et al., 2016).

### Protein processing in the endoplasmic reticulum

We also observed several co-expressed DEGs and DEPs that are associated with the processing of proteins in the endoplasmic reticulum under both HT and DH stress conditions. These genes belonged to the HSP family and included HSP70 (G9639), HSP70-5 (G13246), HSP15.7 (G27775), and HSP17.4B (G39305). HSPs are chaperones that are involved in protein folding and unfolding, both in normal conditions and in response to elevated temperatures (Guo et al., 2014). HSP70 is the most conserved member of the family, in terms of both structure and function (Usman et al., 2015). Lee et al. (2009) observed that HSP70 expression and the development of thermotolerance were significantly associated. HSP70 has also been implicated in thermotolerance in *Oryza sativa* (Sarkar et al., 2013), while HSP70 expression in temperature-tolerant pepper genotypes was found to be higher than in temperature-sensitive varieties, confirming its role in protecting the plant against heat stress (Usman et al., 2017). Here, HSPs in sweetpotato were expressed at higher levels under HT and DH stresses, consistent with the reports on other species.

### Glyoxylate cycle

Two enzymes characteristic of the glyoxylate cycle, namely, isocitrate lyase (ICL) and malate synthase (MS), were found to be induced under all three stress conditions. This suggests that the abiotic stresses activated the glyoxylate cycle to promote the activation of stress-associated proteins in response to adverse environmental conditions. Both ICL and MS have been reported to be induced in the cell senescence (Nishimura and De Bellis, 1991); plant senescence is known to be an important strategy for ensuring species survival under abiotic stress conditions (Gepstein and Glick, 2013). It has also been reported that ICL is involved in tolerance to salt stress, finding that, in rice, the *OsICL* transcript level was increased under salt stress (Yuenyong et al., 2019), whereas ICL activity was decreased in *Pinus pinea* seeds under salt stress (Sidari et al., 2008).

### Starch and cellulose metabolism

We observed that HT, DR, and DH stresses affected the starch synthesis pathway, leading to the down-regulation of G24701 (ADP-glucose pyrophosphorylase beta subunit IbAGPb1A), G35317 (starch branching enzyme I), and G6471 (starch phosphorylase) under HT stress. In addition, the expression and translation levels of G6471 and G48653 (beta-amylase) decreased under DR stress, and both mRNA and protein levels of G6471, G35317, and G48653 were markedly lower in comparison with those of the control under DH stress. These results suggested that starch synthesis may be inhibited under these abiotic stress conditions. Starch synthesis has been found to be reduced under conditions of both temperature and water stress, attributed to the closure of stomata and reduced photosynthetic rates (Zrenner and Stitt, 1991; Thitisaksakul et al., 2012). We also observed that the levels of cellulose degradation-related gene expression increased significantly under HT, DR, or DH stress; these genes included G14317 (PREDICTED: beta-D-xylosidase 1), G27744 (PREDICTED: xylose isomerase), and G9941 (PREDICTED: beta-glucosidase BoGH3B). These genes encode enzymes, and the consequent increase in enzyme activity may be the result of cellulose decomposition by the plant to provide additional carbon sources for the synthesis of secondary metabolites allowing the plant to resist environmental stressors such as HT, DR, and DH. Thus, abiotic stressors can induce the reallocation of carbon for the synthesis of protective molecules involved in osmotic adjustment, protein stabilization, and stress response, among other processes (Zanella et al., 2016; Ribeiro et al., 2022).

### Correlation analysis of co-expressed DEG and DEP genes under HT, DR, and DH stresses

This is the first study investigating the expression of key genes and metabolic pathways responsible for the sweetpotato response to HT, DR, and DH stresses that has used a combination of transcriptomic and proteomic data. The results of the expression patterns and functions of the co-expressed DEGs and DEPs, together with the above discussion, showed that there are various genes associated with the sweetpotato response to HT, DR, and DH stress conditions. These genes included the tocopherol biosynthesis gene *HPD* and *ICL* gene involved in the glyoxylate cycle under all stresses, *HSP* family genes responding to HT and DH stresses as well as the cellulose degradation gene *BoGH3B* under DR and DH stresses. Analysis with Venn diagrams (**Figure 1B**, **Figure 6B**) indicated that some of these genes were only up-regulated under DH stress, including *G9582* (PREDICTED: linoleate 13S-lipoxygenase 2-1, *LOX2.1*) and *G39574* (PREDICTED: clavaminate synthase-like protein, *At3g21360*). Of these, *LOX2.1* is a key gene in the jasmonic acid synthetic pathway that is known to be involved in plant resistance to biological stress conditions (Glauser et al., 2009; Davis et al., 2018). At3g21360 catalyzes amino acids to form hydroxylated amino acids, while hydroxyphenylglycine, (2S, 3R)-3-hydroxyleucine and (2S, 3R)-3-hydroxyphenylalanine are classified as plant defensins (Choroba et al., 2000; Hibi et al., 2015). Both *LOX2.1* and *At3g21360* belong to the subset of plant genes known to be involved in the resistance to biological stress; here, their expression levels were only found to be significantly raised under conditions of DH stress. It is documented that abiotic stress can adversely affect cell viability, which, in turn, influences the expression of defense genes in plants (Zarattini et al., 2021). A number of genes were found to be specifically down-regulated under DH stress, including the growth-related gene *G294* (PREDICTED: adenosine kinase 2-like, *ADK2*), which is involved in the interconversion of cytokinin metabolism. Silencing of *ADK* has been found to lead to impairments in root growth, the appearance of small abnormally shaped leaves, and reduced apical dominance (Schoor et al., 2011). In addition, *G45403* (5-methyltetrahydropteroyltriglutamate–homocysteine methyltransferase, *MHMT*) was uniquely downregulated under DH stress. This gene is involved in gamma-aminobutyric acid (GABA) biosynthesis (Jiao and Gu, 2019), and its expression has been observed to be lowered under various abiotic stress conditions, including salt and heat stress and combinations of the two (Li et al., 2011).

The combined effects of heat and drought stresses are complex, and little is known of their consequences on sweetpotato. Small number of co-expressed DEG and DEP genes and low correlation between mRNA and protein levels in this study for DH were observed. The probable reason might be the low throughput of protein analysis compared to the high-throughput transcriptome analysis. Previous studies have confirmed that some cellular processes, such as posttranscriptional and translational regulation, and different half-lives for mRNAs and proteins, could inhibit correlation between the mRNA and protein levels (Varshavsky, 1996; Grolleau et al., 2002; Becker et al., 2018). It was found that sweetpotato plants exposed to DH stress not only expressed genes and proteins observed to have raised expression under high temperature or drought stress but also showed increased expression of specific genes and proteins associated with molecular regulation. Meanwhile, top 5 up-regulated co-expressed DEGs and DEPs (At5g58770, C24B11.05, Os04g0679100, BACOVA_02659 and HSP70-5) and down-regulated co-expressed DEGs and DEPs (AN3, PMT2, TUBB5, FL and CYP98A3) were identified under DH stress (**Table S7**). The information provided by this study provides a valuable foundation for the influence of DH stress on DEGs/DEPs, which can benefit further research into the mechanism of DH stress tolerance in sweetpotato. This suggests the possibility of enhancing plant tolerance to different stresses through the manipulation of genes and proteins associated with protein stabilization, energy transport, and defense systems against abiotic stress conditions.

## Data availability statement

The transcriptome data are accessible through GEO Series accession number GSE216152 (https://www.ncbi.nlm.nih.gov/geo/query/acc.cgi?acc=GSE216152).

## Author contributions

WT and QL conceived and designed the study; QL and ZL supervised the project; WT and MA performed the experiments and wrote the manuscript; QL and ZL revised the manuscript; ZZ, CL, and MK provided help with the experiments; HY, RG, WS, MM, YZ and XW collected and analyzed the data. All authors contributed to the article and approved the submitted version.
