# Supplementary material for: Comparative transcriptomic and proteomic analysis reveals common molecular factors responsive to heat and drought stresses in sweetpotaoto (Ipomoea batatas)
Source: Front Plant Sci. 2023 Jan 19;13:1081948. doi: 10.3389/fpls.2022.1081948 (PMC9892860; doi:10.3389/fpls.2022.1081948)
Supplement: Supplementary file 1 [file DataSheet_1.docx]

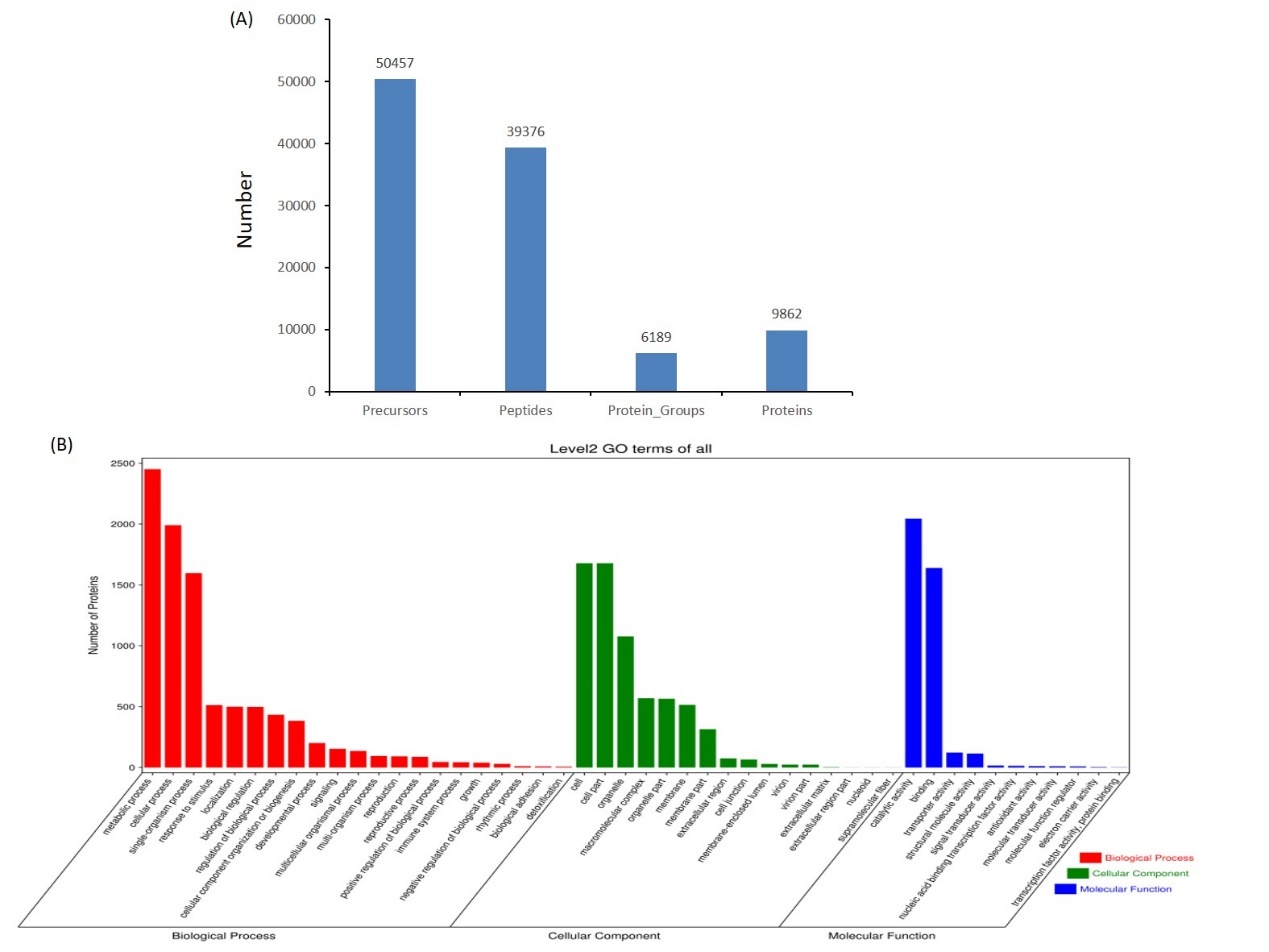


**Figure S1** Proteomic data analysis under HT, DR, and DH stresses. (A) Overview features of proteome sequencing. (B) GO annotation of the identified proteins.


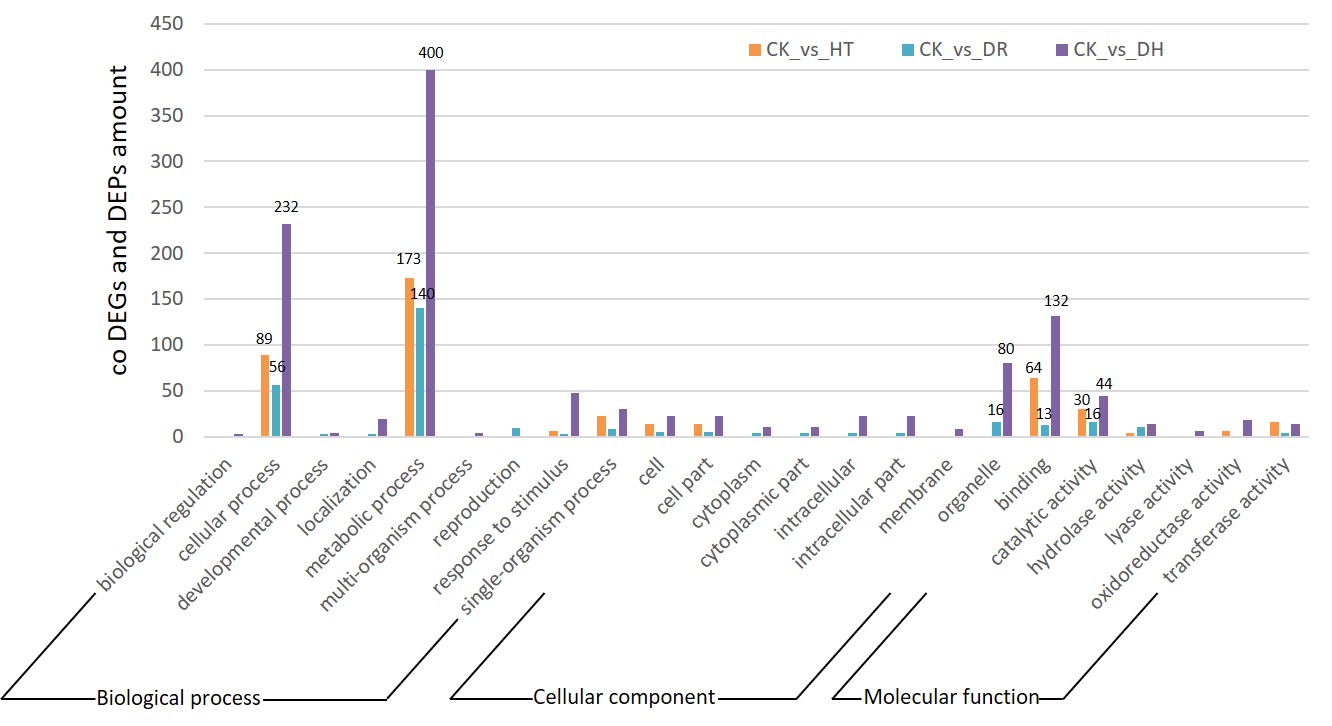


**Figure S2** Gene ontology (GO) classification of co DEGs and DEPs under HT, DR, and DH stresses. HT, heat; DR, drought; DH, drought and heat stresses.


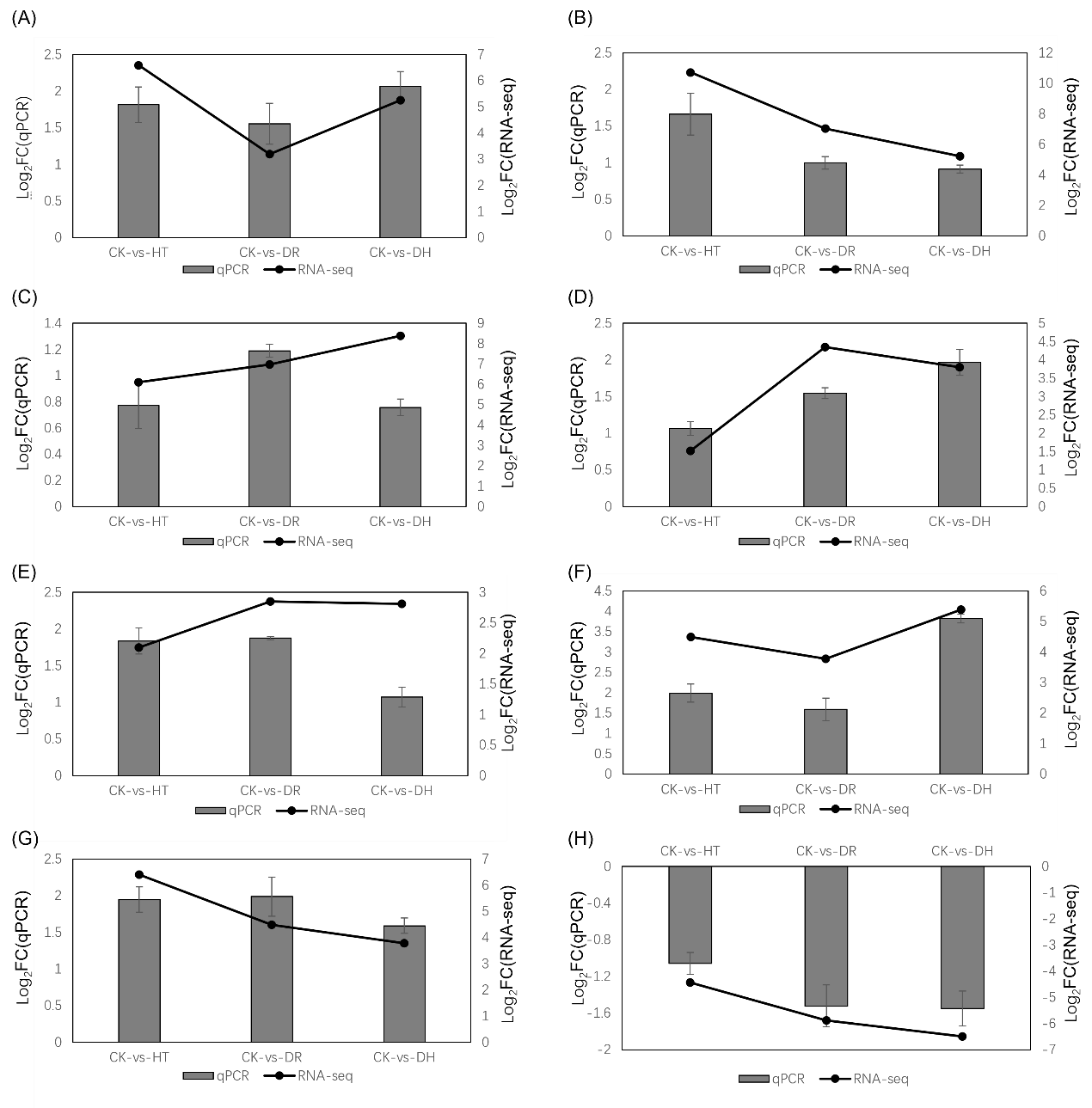


**Figure S3** qRT-PCR confirmation of RNA-seq results. Co-expressed DEG and DEP genes (G13246 (*Hsp70-5*), G14317 (*BXL1*), G23318 (*ICL*), G27744 (*XYLA*), G33304 (*PHOS34*), G38888 (*HPD*), G42670 (*MLS*), G26280 (*CHLH*)) expression levels and DEG values are shown at (A), (B), (C), (D), (E), (F), (G), and (H), respectively. Expression values determined by qRT-PCR are presented as columns and RNA-seq FC are presented as curves. Standard error bars are showed for the expression values determined by qRT-PCR. HT, heat; DR, drought; DH, drought and heat stresses.
